# West Nile Virus Spreads Transsynaptically within the Pathways of Motor Control: Anatomical and Ultrastructural Mapping of Neuronal Virus Infection in the Primate Central Nervous System

**DOI:** 10.1371/journal.pntd.0004980

**Published:** 2016-09-12

**Authors:** Olga A. Maximova, John G. Bernbaum, Alexander G. Pletnev

**Affiliations:** 1 Laboratory of Infectious Diseases, National Institute of Allergy and Infectious Diseases, National Institutes of Health, Bethesda, Maryland, United States of America; 2 Office of the Chief Scientist, Integrated Research Facility, National Institute of Allergy and Infectious Diseases, National Institutes of Health, Frederick, Maryland, United States of America; Molecular Biology Unit (MBU), INDIA

## Abstract

**Background:**

During recent West Nile virus (WNV) outbreaks in the US, half of the reported cases were classified as neuroinvasive disease. WNV neuroinvasion is proposed to follow two major routes: hematogenous and/or axonal transport along the peripheral nerves. How virus spreads once within the central nervous system (CNS) remains unknown.

**Methodology/Principal Findings:**

Using immunohistochemistry, we examined the expression of viral antigens in the CNS of rhesus monkeys that were intrathalamically inoculated with a wild-type WNV. The localization of WNV within the CNS was mapped to specific neuronal groups and anatomical structures. The neurological functions related to structures containing WNV-labeled neurons were reviewed and summarized. Intraneuronal localization of WNV was investigated by electron microscopy. The known anatomical connectivity of WNV-labeled neurons was used to reconstruct the directionality of WNV spread within the CNS using a connectogram design. Anatomical mapping revealed that all structures identified as containing WNV-labeled neurons belonged to the pathways of motor control. Ultrastructurally, virions were found predominantly within vesicular structures (including autophagosomes) in close vicinity to the axodendritic synapses, either at pre- or post-synaptic positions (axonal terminals and dendritic spines, respectively), strongly indicating transsynaptic spread of the virus between connected neurons. Neuronal connectivity-based reconstruction of the directionality of transsynaptic virus spread suggests that, within the CNS, WNV can utilize both anterograde and retrograde axonal transport to infect connected neurons.

**Conclusions/Significance:**

This study offers a new insight into the neuropathogenesis of WNV infection in a primate model that closely mimics WNV encephalomyelitis in humans. We show that within the primate CNS, WNV primarily infects the anatomical structures and pathways responsible for the control of movement. Our findings also suggest that WNV most likely propagates within the CNS transsynaptically, by both, anterograde and retrograde axonal transport.

## Author Summary

West Nile virus (WNV) is a mosquito-borne neurotropic flavivirus that has emerged as a human pathogen of global scale. During recent WNV outbreaks in the US, half of the reported human cases were classified as neuroinvasive disease. Although much research has been done, there are still gaps in our understanding of WNV neuropathogenesis. While WNV neuroinvasion is proposed to occur by the hematogenous route and/or by axonal transport along the peripheral nerves, how virus spreads once within the central nervous system (CNS) remains unknown. In this study, we examined the expression of viral antigens in the CNS of monkeys that were intrathalamically inoculated with WNV. Next, we mapped the localization of WNV-infected neurons to specific anatomical structures, identified the intraneuronal localizations of WNV particles and investigated the role of neuronal connectivity in the spread of WNV within the CNS. Our results revealed that all structures containing WNV-labeled neurons belonged to the pathways of motor control. Virions were found in close vicinity to the axodendritic synapses, strongly indicating transsynaptic spread of the virus. Neuronal connectivity-based reconstruction of the directionality of transsynaptic virus spread suggests that, within the CNS, WNV can utilize both anterograde and retrograde axonal transport to infect connected neurons.

## Introduction

West Nile virus (WNV) is a mosquito-borne neurotropic flavivirus that has emerged as a human pathogen of global scale [1]. During the latest and largest outbreak of human WNV disease in US history during 2012–2013 [2,3], over half of the reported cases (51%) were classified as WNV neuroinvasive disease. Although much research has been done, there are still gaps in our understanding of WNV neuropathogenesis [4]. There is no consensus on how WNV infects the central nervous system (CNS). Two hypotheses of neuroinvasion are being considered: (i) hematogenous route and (ii) transneural entry through peripheral nerves. However, regardless of the mode of virus entry from the periphery, it is not clear how virus spreads once within the CNS.

Cell-to-cell spread of viruses contributes significantly to the pathogenesis of viral infections by facilitating virus dissemination and immune evasion [5]. The ability of several neurotropic viruses to spread between neurons via neuronal synapses and use axonal transport (anterograde, retrograde, or both) is well recognized and has been successfully exploited to trace neuronal connectivity. Of these viruses, the most studied are the alpha herpes viruses and rabies virus (reviewed in [6–9]). A study using an *in vitro* system of compartmentalized neuronal cultures showed that WNV can spread between neurons in both anterograde and retrograde directions via axonal transport [10]. However, how WNV spreads *in vivo*, especially within the CNS, is less clear. Transneuronal WNV spread was reported as a putative route of neuroinvasion after sciatic nerve inoculation in hamsters [10,11], but the brain was not studied in this model, and it was speculated that in the CNS, both anterograde and retrograde neuronal transport contributes to “centrifugal” spread of WNV among neurons in the brain [10]. It is also not clear how well rodent models reproduce all aspects of WNV neuropathogenesis in humans [4]. Nonhuman primates (NHP) represent a more suitable model due to their natural susceptibility to a wide range of human pathogens and the high degree of genetic similarity to humans [12]. However, primates do not develop neurological WNV disease after peripheral (either natural or experimental) infection [13–17]. On the other hand, the NHP model of neuroinfection, in which animals are inoculated intracerebrally, remarkably recapitulates the features of WNV encephalomyelitis seen in humans [15–19].

In humans with WNV neuroinvasive disease, the CNS structures that are often involved include (listed by an increasing gradient of the severity of infection): cerebral cortex (least severe), basal ganglia, thalamus, brainstem, cerebellum, and spinal cord (most severe) [20–24]. The possibility of WNV propagation via neuronal processes within the CNS was suggested from autopsy findings in an immunosuppressed patient with a fatal WNV encephalitis [25]. However, the connectivity between affected structures and its possible role in virus spread within the CNS have not been studied. Here, we used the CNS tissues from our previous NHP study [19], in which animals were inoculated intrathalamically with WNV, and investigated the role of the neuroanatomical connectivity in the spread of WNV within the brain and spinal cord.

## Materials and Methods

### Ethics statement

The rhesus macaques (*Macaca mulatta*) used for this study were housed in a BSL-3 facility in compliance with the National Institute of Allergy and Infectious Diseases (NIAID), Division of Intramural Research (DIR) Animal Program Policy on Social Housing of Non-Human Primates, and Comparative Medicine Branch NHP enrichment programs. Animals were provided with commercial food pellets supplemented with appropriate treats. Drinking water was provided *ad libitum*. All steps were taken to minimize suffering. The experimental procedures requiring anesthesia were performed using ketamine hydrochloride or other anesthetics at the discretion of the attending veterinarian. For euthanasia, ketamine hydrochloride pre-anesthesia and sodium pentobarbital were used. The NIAID DIR Animal Care and Use Committee approved the animal study proposal (#LID 7E). The NIAID DIR Animal Care and Use Program, as part of the NIH Intramural Research Program (IRP), complies with all applicable provisions of the Animal Welfare Act (http://www.aphis.usda.gov/animal_welfare/downloads/awa/awa.pdf) and other Federal statutes and regulations relating to animals. The NIAID DIR Animal Care and Use Program is guided by the "U.S. Government Principles for the Utilization and Care of Vertebrate Animals Used in Testing, Research, and Training" (http://oacu.od.nih.gov/regs/USGovtPrncpl.htm). The NIAID DIR Animal Care and Use Program acknowledges and accepts responsibility for the care and use of animals involved in activities covered by the NIH IRP’s PHS Assurance #A4149-01, last issued 11/24/2014. As partial fulfillment of this responsibility, the NIAID DIR Animal Care and Use Program ensures that all individuals involved in the care and use of laboratory animals understand their individual and collective responsibilities for compliance with that Assurance, as well as all other applicable laws and regulations pertaining to animal care and use. The NIAID DIR Animal Care and Use Program has established and will maintain a program for activities involving animals in accordance with the most recent (2011, 8th edition) of “The Guide for the Care and Use of Laboratory Animals” (ILAR, NRC) (http://oacu.od.nih.gov/regs/guide/guide_2011.pdf). The policies, procedures and guidelines for the NIH IRP are explicitly detailed in NIH Policy Manual 3040–2, “Animal Care and Use in the Intramural Program” (PM 3040–2) and the NIH Animal Research Advisory Committee Guidelines (ARAC Guidelines). Those documents are posted on the NIH Office of Animal Care and Use public website at: http://oacu.od.nih.gov.

### Virus and animal tissue samples

Our animal model of WNV neuropathogenesis in NHPs (*Macaca mulatta*; WNV-seronegative; 2–3 year old) that were inoculated intrathalamically (bilaterally) with a dose of 5.0 log_10_ PFU of wild-type WNV strain NY99-35262 (hereafter WNV) has recently been described [19]. We performed a systematic collection of all major CNS regions from these animals for downstream analyses. In this study, CNS tissues were examined by immunohistochemistry and electron microscopy. CNS tissues were from twelve WNV-infected monkeys (3 days post infection (dpi) [n = 3]; 7 dpi [n = 3]; and 9/10 dpi (9 dpi [n = 5]; 10 dpi [n = 1]) and two mock-inoculated monkeys (7 dpi [*n* = 1] and 10 dpi [*n* = 1]). WNV-infected animals developed a fulminant encephalomyelitis by 9/10 dpi (details of the clinical course, CNS virus burden, and histopathological scores can be found in our prior publication [19]). Brains and spinal cords were collected immediately after euthanasia and cardiac perfusion with sterile saline. After a parasagittal cut, the right brain hemisphere was fixed in 10% buffered formalin. Rhesus Mon-key Brain Matrix (Ted Pella, Redding, CA) was used to make 4 mm coronal brain slices that were further cut to facilitate mounting of subsequent sections onto standard 1 x 3 inches slides. Slices were routinely processed and embedded in paraffin. Two 5 μm sections (1st and 4th) from each paraffin block were mounted onto single slides and processed for immunohistochemistry. Spinal cord was dissected transversely and sections from cervical, thoracic, and lumbar regions were mounted onto single slides and also processed for immunohistochemistry.

### Immunohistochemistry

Immunohistochemical detection of WNV antigens in the CNS of rhesus monkeys was performed using WNV-specific primary antibodies in hyperimmune mouse ascitic fluid (ATCC VR-1267 AF; 1:1000) and subsequent steps were according to previously described procedures [26]. Diaminobenzidine was used for colorimetric detection of WNV antigens. Sections were counterstained with hematoxylin.

### Digital pathology and neuroanatomical mapping

Whole tissue section imaging was performed at 20x magnification using ScanScope XT (Aperio, Vista, CA). Aperio Spectrum Plus and ImageScope software was used for digital slide organization, viewing, and analysis. We analyzed all major CNS regions including: cerebral cortex, basal ganglia, thalamus, midbrain, pons, medulla oblongata, cerebellum (cerebellar cortex and deep cerebellar nuclei), and spinal cord (cervical, thoracic, and lumbar regions). The “Primate Brain Maps: Structure of the Macaque Brain” [27] were used for neuroanatomical orientation and mapping. To examine the WNV-immunoreactivity and to add to the visualization of WNV-antigen positive cells in the cerebellar cortex, a custom “WNV-labeled cell segmentation” image analysis algorithm was developed based on the ImageScope nuclear algorithm.

### Electron microscopy

For ultrastructural analysis, core tissue samples (2 mm in diameter; 4 mm thick) were extracted using sterile Harris Uni-Cores (Ted Pella, Redding, CA). Samples that included the gray matter (wherever possible) were extracted from the following CNS regions: cerebral cortex, basal ganglia, thalamus, pons, medulla oblongata, cerebellar cortex, and spinal cord (cervical and lumbar regions). For the cerebellar cortex, core samples were extracted from the folia in a manner that included the molecular layer, Purkinje cell layer, and granule cell layer. For the spinal cord, core samples were extracted from the ventral horns.

Collected core tissue samples were fixed in 2.5% glutaraldehyde and 2% paraformaldehyde (Electron Microscopy Sciences, Hatfield, PA), then washed in Millonig’s sodium phosphate buffer (Tousimis Research, Rockville, MD), post-fixed in 1% osmium tetroxide (Electron Microscopy Sciences), stained *en bloc* with 2% uranyl acetate (Fisher Scientific, Waltham, MA), dehydrated in increasing concentrations of ethanol, and then infiltrated and embedded in Spurr plastic resin (Electron Microscopy Sciences). Embedded tissue samples were sectioned using a Leica UC7 Ultramicrotome (Leica Microsystems, Buffalo Grove, IL). Ultra-thin sections (60–80 nm in thickness) were collected, mounted onto 200 mesh copper grids, and contrasted with lead citrate (Fisher Scientific). The grids were then examined and imaged using a transmission electron microscope (FEI G2 Tecnai).

### Design of connectograms for visualization of neuroanatomical connectivity and reconstruction of virus spread

The method of circular representation, named a “connectogram”, is an intuitive and suitable approach for the visualization and interpretation of neuroanatomical connectivity using magnetic resonance imaging [28,29]. This type of representation is also highly suitable for visualization of complex neuroanatomical connections with an attempt to reconstruct virus spread between the infected CNS structures in this study. For this purposes, we adopted the connectogram idea and manually created our connectograms using Adobe Illustrator. The information used to create the connectograms is based on the literature review of established connectivity only between neuroanatomical structures relevant to this study.

## Results and Discussion

### Mapping anatomical localization of WNV within the CNS

Our first goal was to identify WNV-labeled cells using immunohistochemistry and then map their distribution to specific anatomical structures within the CNS. We did not detected WNV antigens at 3 dpi in any CNS region. WNV-labeled neurons became readily detectable in the CNS at 7 dpi and 9/10 dpi. WNV-infected CNS regions, anatomical structures/types of neurons, reference virus titers [19], extent/intensity and timing of WNV-labeling, as well as references to representative images in this report are summarized in Table 1. A general pattern of anatomical localization and extent of neuronal WNV-labeling closely followed the distribution and amounts of infectious virus at the same time points. To add to these comparisons, the changes in WNV loads within each major CNS region during the time course of neuroinfection are provided by radar charts in S1 Fig. One caveat of this study is the fact that we were unable to detect WNV antigens by immunohistochemistry at 3 dpi. This could be explained by the immunohistochemical limit of flavivirus detection of approximately 3 log_10_ PFU/g (personal observation and compare mean virus titers and average WNV-labeling in Table 1). Whether other neuronal cells could be infected at the levels below our limit of detection remains an open question.

**Table 1:** Summary of CNS region names, structures/types of neurons, reference virus titers, extent/intensity, timing of neuronal WNV-labeling, and references to representative images of WNV-labeled neurons.

CNS region	Virus titer^a^ (log_10_PFU/g)			Structure/type of neurons	Average WNV-labeling^b^			Representative images
	3 dpi	7 dpi	9/10 dpi		3 dpi	7 dpi	9/10 dpi	
MOTOR CORTEX	1.8±0.1	2.9±0.3	1.8±0.1	Corticospinal motor neurons	**-**	**++**	**-**	Fig 1
SUBCORTICAL REGIONS	3.0±0.5	5.1±0.1	3.5±0.4	Motor thalamus	**-**	**+++**	**+**	Fig 2A
	2.4±0.4	4.1±0.1	2.1±0.3	Basal ganglia	**-**	**+**	**-**	Fig 2B
MIDBRAIN	2.1±0.3	4.5±0.3	2.5±0.3	Substantia nigra pars compacta	**-**	**++**	**+**	Fig 2C
				Red nucleus magnocellular	**-**	**-**	**+**	Fig 2D
PONS/ MEDULLA OBLONGATA	1.8±0.2	5.1±0.1	4.9±0.2	Pontine nuclei	**-**	**+**	**++**	Fig 2E
				Vestibular nuclei	**-**	**-**	**++**	Fig 2F
				Medullary reticular formation	**-**	**+**	**++**	Fig 2G
				Inferior olivary nuclei	**-**	**+**	**++**	Fig 2H
				Accessory cuneate nucleus	**-**	**-**	**++**	Fig 2I
CEREBELLUM	1.8±0.2	6.4±0.3	6.9±0.1	Deep cerebellar nuclei	**-**	**+**	**+++**	Fig 3
				Purkinje cells	**-**	**++**	**+++**	Fig 4A and S2 Fig
				Granule cells	**-**	**-**	**+**	Fig 4C and 4D
SPINAL CORD: C^c^	≤1.7	5.7±0.2	5.7±0.2	Spinal motor neurons	**-**	**++**	**+++**	Fig 5A and 5D
SPINAL CORD: T^d^	≤1.7	5.6±0.3	5.6±0.1	Spinal motor neurons	**-**	**++**	**++**	Fig 5B and 5E
SPINAL CORD: L^e^	≤1.7	5.6±0.4	5.8±0.2	Spinal motor neurons	**-**	**++**	**+++**	Fig 5C and 5F
				Clarke’s column (C8 –L3)^f^	**-**	**-**	**+++**	Fig 5E

^a^ Refers to previously reported virus titers from the left hemisphere [19]. Limit of detection: 1.7 log_10_PFU/g of tissue.

^b^ The presence or absence of neuronal labeling is indicated by (+) or (-), respectively. Average extent and intensity of labeling is indicated by the number of (+) as follows: +, minimal (light gray shading); ++, moderate (medium gray shading); +++, maximal (dark gray shading).

^c^ C—cervical;

^d^ T—thoracic;

^e^ L—lumbar;

^f^ Clarke’s column is found in segments C8—L3 of the spinal cord.

**Fig 1:**
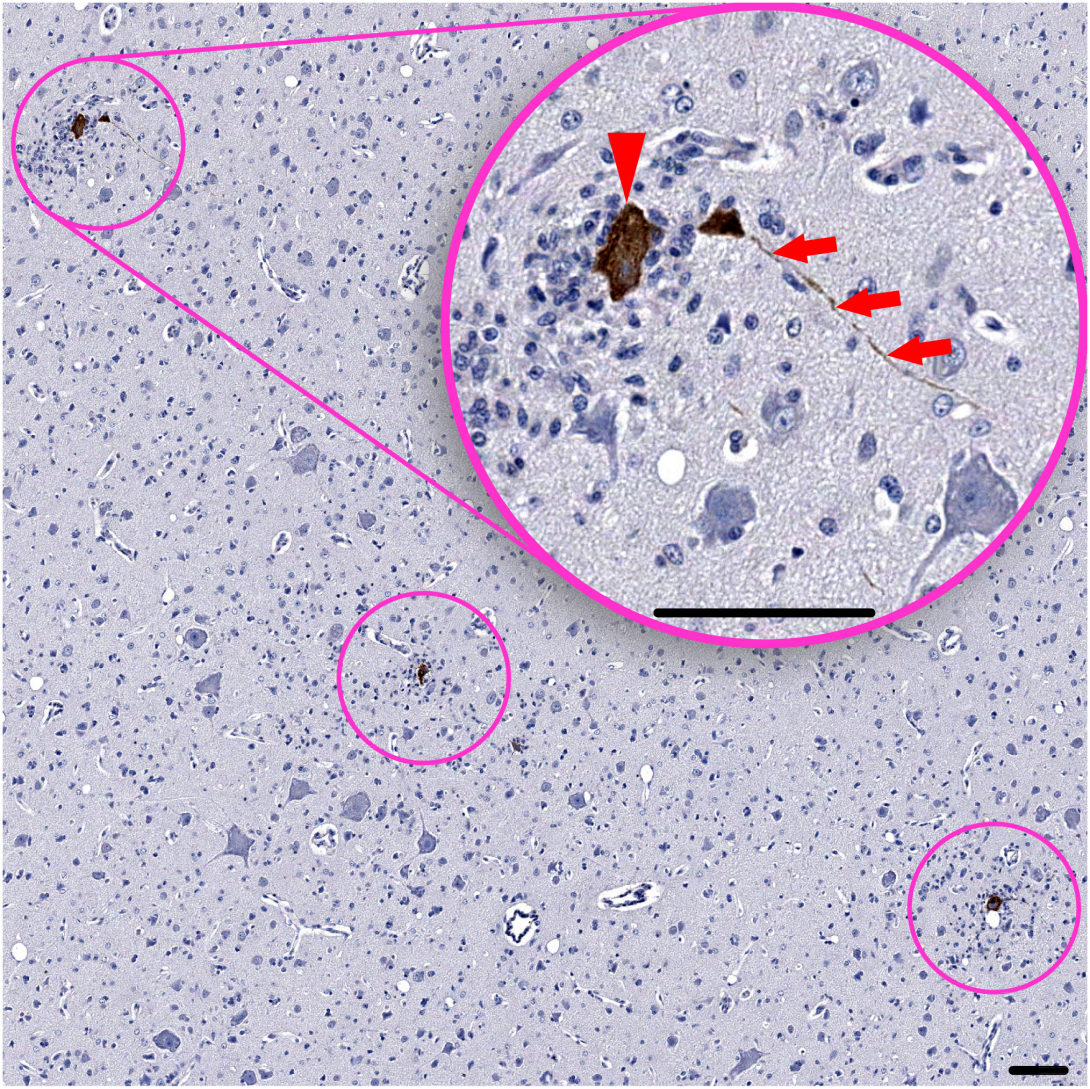
WNV-labeled corticospinal motor neurons in the cortical layer 5 of the primary motor cortex. Inset shows one of the circled areas at higher magnification. Red arrowhead points to a large WNV-labeled corticospinal motor neuron (Betz cell). Note that all three circled WNV- labeled Betz cells are damaged and undergoing neuronophagia (7 dpi). Also note an adjacent long axonal profile that contains WNV-antigens (red arrows). Scale bars: 100 μm.

**Fig 2:**
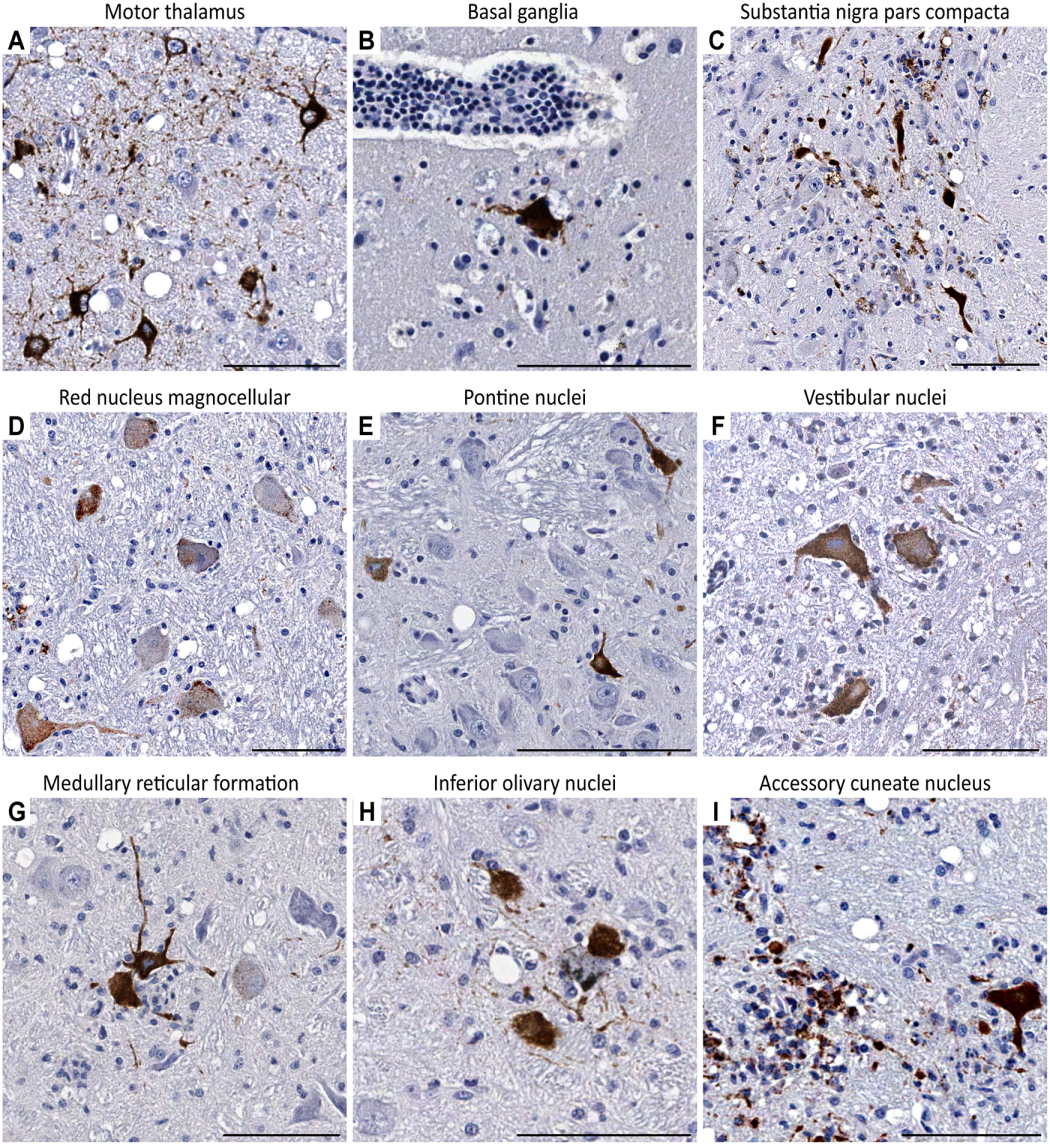
WNV-labeled neurons in the subcortical structures, midbrain, pons, and medulla oblongata. (**A**) Neurons in the motor thalamus display intense WNV-labeling in the neuronal perikarya and dendritic/axonal profiles (7 dpi). (**B**) WNV-labeled neuron in the basal ganglia. Note its close vicinity to a perivascular inflammatory infiltrate (7 dpi). (**C**) WNV-labeled neurons in the substantia nigra pars compacta (9 dpi). (**D**) Red nucleus magnocellular part (9 dpi). Perikarya of large neurons display granular WNV-labeling. Also note WNV-labeled neuritic profile emanating from the infected neuron in the lower-left corner of the field. Representative images of WNV-labeled neurons in the brainstem are shown in **E** (pontine nuclei; 7 dpi), **F** (vestibular nuclei; 9 dpi), **G** (medullary reticular formation; 7 dpi), **H** (inferior olivary nuclei; 7 dpi), and **I** (accessory cuneate nucleus; 9 dpi). Scale bars: 100 μm.

WNV-labeling was detected in the neuronal cytoplasm and processes of the following anatomical structures and/or neuronal types: motor cortex (corticospinal motor neurons [Betz cells]); subcortical regions (neurons in the motor [ventrolateral] thalamus and basal ganglia); midbrain (substantia nigra pars compacta and red nucleus magnocellular); and pons/medulla oblongata (pontine nuclei, vestibular nuclei, medullary reticular formation, inferior olivary nuclei, and accessory cuneate nucleus) (Figs 1 and 2).

**Fig 3:**
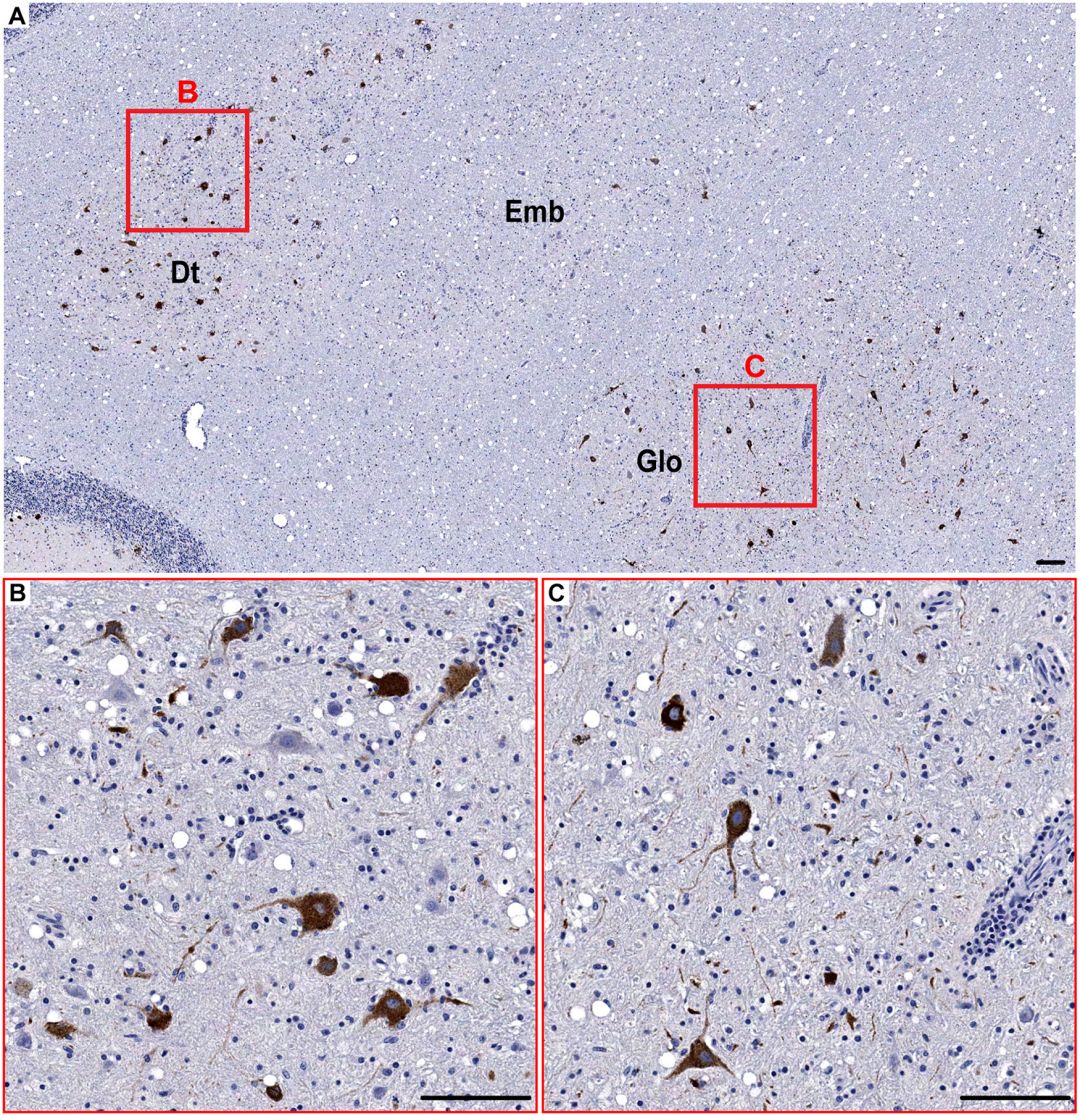
WNV-labeled neurons in the deep cerebellar nuclei. (**A**) Low magnification overview image showing many WNV-labeled neurons in the dentate nucleus (Dt), emboliform nucleus (Emb), and globose nucleus (Glo) (9 dpi). **B** and **C** show corresponding boxed areas at higher magnification. Scale bars: 100 μm.

**Fig 4:**
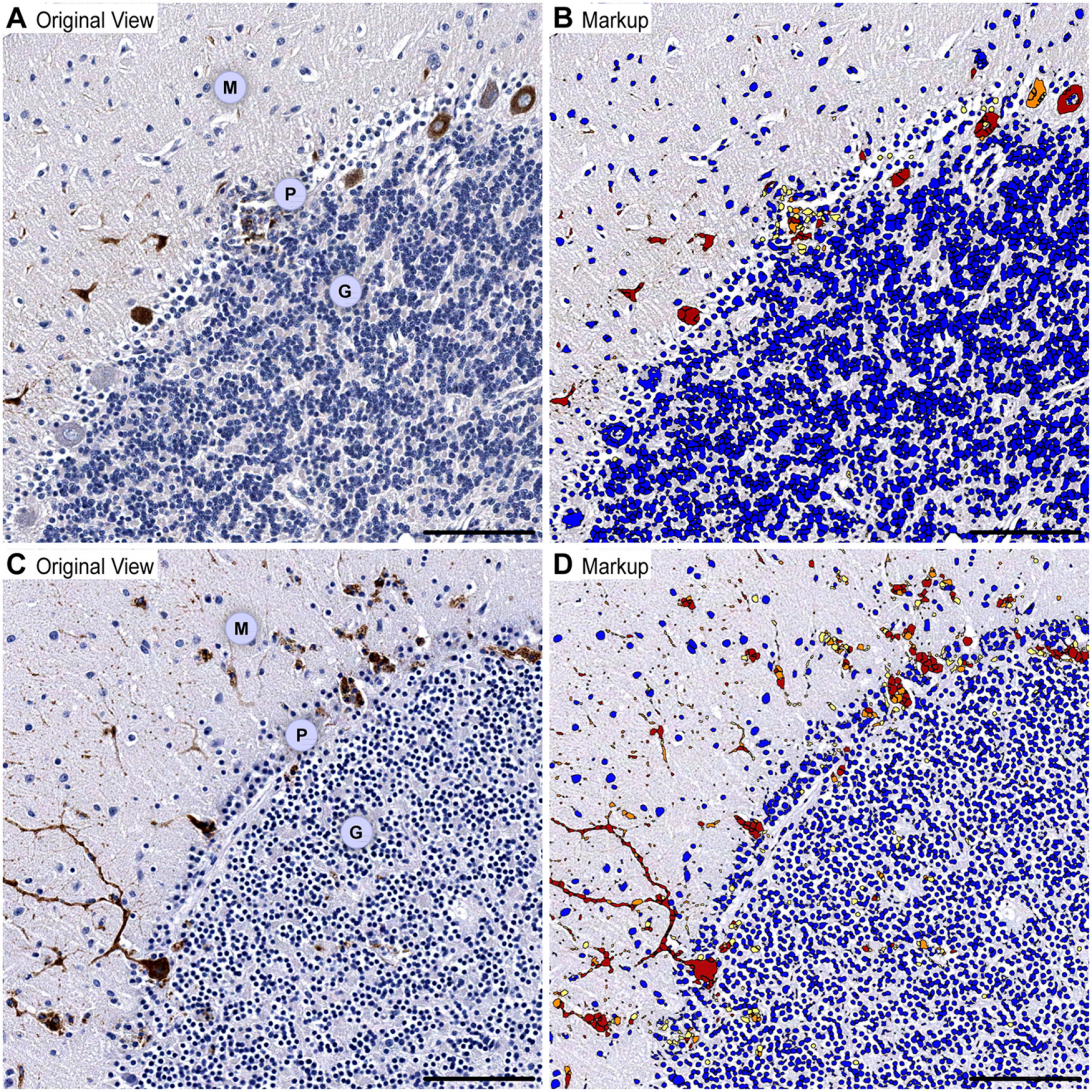
WNV-labeled neurons in the cerebellar cortex. Original views of WNV-labeled neurons at 7 dpi (**A**) and 9 dpi (**C**). **B** and **D** show the corresponding markup images of **A** and **C** after applied “WNV-labeled cell segmentation” image analysis algorithm (WNV immunoreactivity: red, strong; orange, moderate; yellow, weak; negative, blue). Note an increasing WNV immunoreactivity in the somatodendritic compartments of Purkinje cells from 7 dpi to 9 dpi (compare **B** and **D**) and variable immunoreactivity in small groups of granule neurons only at 9 dpi (**D**). M, molecular layer; P, Purkinje cell layer; G, granule cell layer. Scale bars: 100 μm.

In the cerebellum, WNV-labeling was unambiguously detected in neurons of the deep cerebellar nuclei (Fig 3) and in the Purkinje cells (Fig 4 and S2 Fig). WNV-labeling of the granule neurons was much less frequent. To better appreciate the differences in WNV immunoreactivity between the infected cells of the cerebellar cortex, we developed a custom “WNV-labeled cell segmentation” image analysis algorithm, which produced markup images highlighting the intensity of WNV immunoreactivity in the Purkinje cells (Fig 4B and 4D) and granule neurons (Fig 4D).

Of note, despite the fact that only a few small groups of granule neurons were infected, we observed a substantial focal rarefaction of the granule cell layer at 9 dpi. This phenomenon cannot be explained by virus-induced cell death since only a small number of these cells were WNV-positive, nor can it be ascribed to a known artefact of granule cell dissolution due to postmortem autolysis. The latter is because necropsy and tissue fixation were performed immediately after euthanasia and also because our experiments were well controlled by inclusion of mock-inoculated animals that were euthanized at the same time points as WNV-infected animals (compare anti-NeuN immunostaining highlighting the rarefaction of the granule cell layer in WNV-infected animals and normal granule cell layer in mock-inoculated animals, S4 Fig). This phenomenon therefore remains unexplained and deserves further investigation.

**Fig 5:**
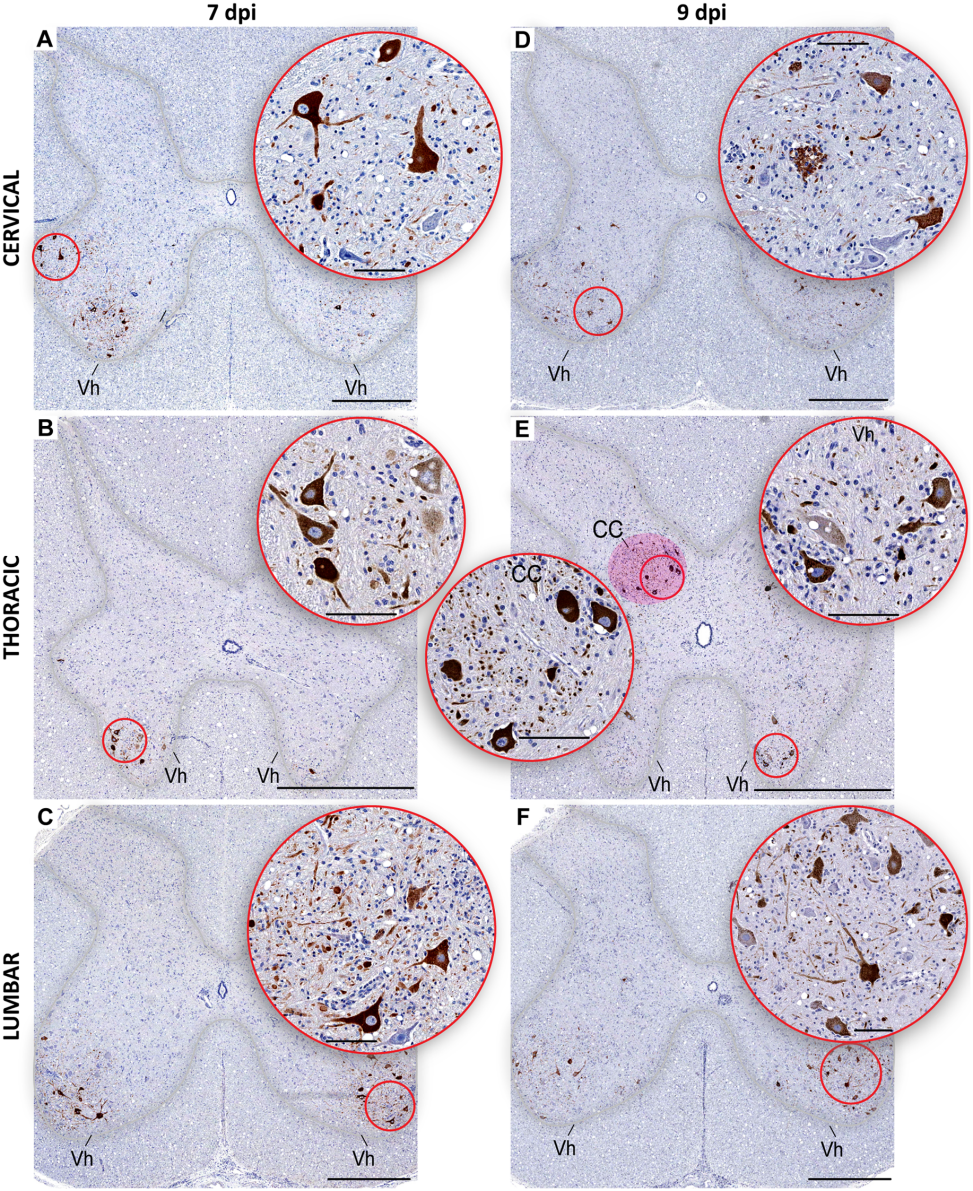
WNV-labeled neurons in the spinal cord. Representative images of WNV-labeled neurons in the cervical (**A** and **D**), thoracic (**B** and **E**), and lumbar regions (**C** and **F**) of the spinal cord are shown on indicated dpi. Approximate boundaries of the spinal cord gray matter are outlined in the overview images. Clarke’s column is shown by magenta overlay in **E**. Round insets show the corresponding circled areas at higher magnification. Note that the majority of WNV-labeled neurons occupy a medial portion of the Clarke’s column. Vh, ventral horns; CC, Clarke’s column. Bars in overview images: 1000 μm. Bars in the round insets: 100 μm.

In the spinal cord, WNV-labeling was detected in the lower motor neurons residing in the *Rexed’s* laminae IX of the ventral horns, spanning cervical, thoracic, and lumbar regions (Fig 5). However, in addition to this, an intriguing finding was that many spinocerebellar relay neurons that occupy a discrete nucleus known as Clarke’s column also contained large amounts of WNV antigens in their cytoplasm and transverse axonal profiles (Fig 5E).

These mapping results, when taken together, provided a detailed picture of WNV infection of the CNS. Interestingly, all structures identified as harboring WNV-labeled neurons are thought to participate in the control of movement (Table 2)

**Table 2:** Summary of neurological functions related to the structures containing WNV-labeled neurons.

CNS region	WNV-infected structure or type of neurons	Functions	References
Motor cortex	Corticospinal motor neurons	Planning, initiating, and directing complex voluntary movement	[30–32]
Subcortical regions/Midbrain	Motor thalamus	Acts as an integrator of motor information; maintaining posture, general movements and motor learning	[30,33,34]
	Basal ganglia	Selection and initiation of most appropriate motor or behavioral programs	[30,35–37]
	Substantia nigra pars compacta		
	Red nucleus magnocellular	Activity correlating with duration, amplitude, and velocity of independent distal movements	[30,38–40]
Pons	Pontine nuclei	Relay signals from the motor cortex to the cerebellar cortex	[30]
Medulla oblongata	Vestibular nuclei	Integration and encoding of self-motion for motor control	[30,41]
	Medullary Reticular Formation	Control of posture during ongoing movements	[30,42]
	Inferior olivary nuclear complex	Motor learning and motor timing	[30,43,44]
	Accessory cuneate nucleus	Relays proprioceptive signals to the cerebellum	[30]
Cerebellum	Deep cerebellar nuclei	Modulation of movement; detection of motor errors and mediation of reduction in the error (motor learning)	[30,45]
	Purkinje cells		
	Granule cells		
Spinal cord	Spinal motor neurons	Initiation of skeletal muscle contraction and movement	[30]
	Clarke’s column	Relays proprioceptive signals to the cerebellum and integrates corticospinal inputs with relevance to motor planning and evaluation	[30,45,46]

Of note, during the terminal stage of neuroinfection, WNV also infected the structures that relay proprioceptive signals from the upper parts of the body (accessory cuneate nucleus) and from the lower parts of the body (Clarke’s column) to the cerebellum. Clarke’s column (medial portion) also integrates the corticospinal inputs with relevance to motor planning and evaluation [46].

Remarkably, all WNV-infected structures and/or neuronal groups identified in this study were also reported to be affected in humans with WNV encephalomyelitis (i.e., cerebral cortex, basal ganglia, thalamus, substantia nigra, red nucleus, pons, vestibular nuclei, medulla, inferior olive, cuneate nucleus, Purkinje cells, dentate nucleus, Clarke’s column, and ventral horns of spinal cord [20–25,47–51]. Our findings are also in line with the pioneering studies of WNV encephalitis in intracerebrally inoculated nonhuman primates [15–18]. These early studies, although not well equipped to precisely detect specific groups of infected neurons, clearly showed infection of brainstem, cerebellum and spinal cord. Our findings provide the evidence that, within the primate CNS, WNV preferentially infects specific neuroanatomical structures responsible for the control of movement.

### Identification of intraneuronal localizations of WNV

We next used electron microscopy (EM) to determine intraneuronal localization of WNV particles. It is generally accepted that in order to detect virus particles in tissue culture by electron microscopy (EM), the virus titers have to be high (i.e., 10^5^ to 10^6^ particles per milliliter) [52]. The same is true for detection of viruses in tissues. A major limitation of virus detection in tissues by EM is that the sampling might miss the areas containing viruses. With this in mind, we focused on the most heavily infected CNS regions with highest virus loads (i.e., cerebellum and spinal cord; see Table 1) from animals which developed fulminant encephalitis (at 9/10 dpi). In order to maximize the probability of virion detection, we used a targeted small-volume sampling of the gray matter for the ultrastructural analysis (see Materials and Methods). Tissue-core samples of the cerebellar cortex included the molecular layer, Purkinje cell layer, and granule cell layer. Tissue-core samples of the spinal cord contained the gray matter from the ventral horns.

Ultrastructural analysis confirmed WNV infection of the Purkinje cells in the cerebellar cortex and motor neurons in the ventral horns of the spinal cord. We also examined many other CNS regions with lower virus burden (i.e., cerebral cortex, basal ganglia, thalamus, pons, and medulla oblongata) but, as expected, were unable to detect virions.

**Fig 6:**
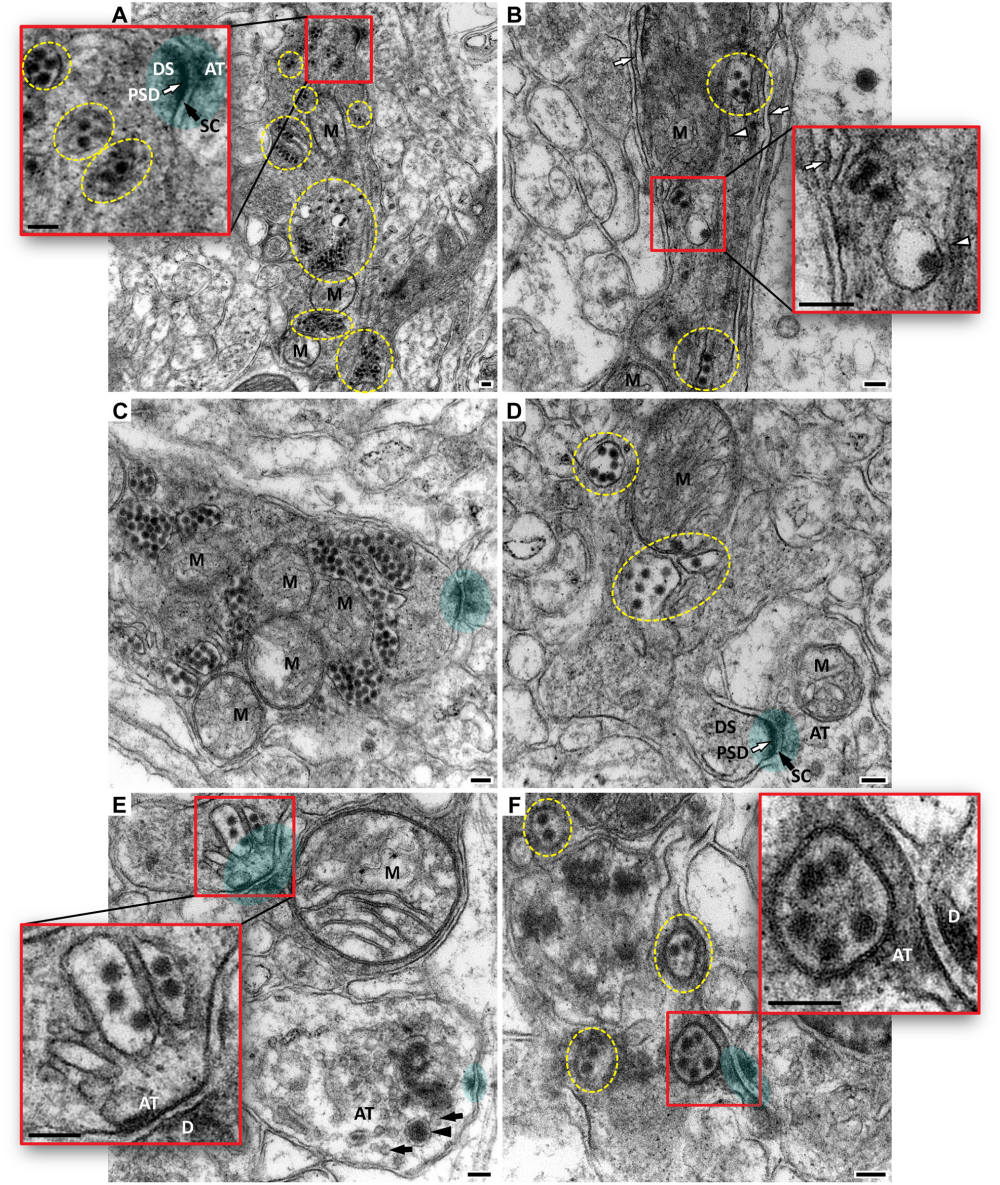
Ultrastructural localization of WNV particles in the cerebellar cortex. Shown are electron microscopy images of dendritic arbors of Purkinje cells (PCs). Some asymmetric axo-dendritic synapses are shown by green overlays. In panels (**A** and **D**): white arrows point to the postsynaptic density (PSD) and black arrows show synaptic clefts (SC). In some panels the groups of virions are outlined by yellow dashed circles/ovals. Insets in (**A**, **B**, **E**, and **F**) show the corresponding red boxed areas at higher magnification. (**A**) Bifurcating PC dendrite contains multiple spherical electron-dense virions. Inset shows several virions in close vicinity to the dendritic spine (DS) which is synapsing with axonal terminal (AT). (**B**) A shaft of another PC dendrite is shown at higher magnification. Virions can be seen in association with the hypolemmal cisternae of the agranular endoplasmic reticulum (AER; white arrows) and microtubule structures (white arrowheads). Inset shows three virions adjacent to the AER (white arrow) and one virion inside the vesicle adjacent to microtubules covered with a “cottony” material. (**C** and **D**) Virions within PC dendrites are found in close vicinity to synapses. (**E**) Virion-containing vesicles are in the presynaptic position in the axonal terminal. Another axonal terminal in the lower right corner contains synaptic vesicles (black arrows) and one large dense-core vesicle (black arrowhead). (**F**) Virions are enclosed within the autophagosome-like vesicles with double-layered membrane. Inset shows one such vesicle at presynaptic position (~40 nm away from the active zone of the synapse). M, mitochondria. Scale bars: 100 nm.

**Fig 7:**
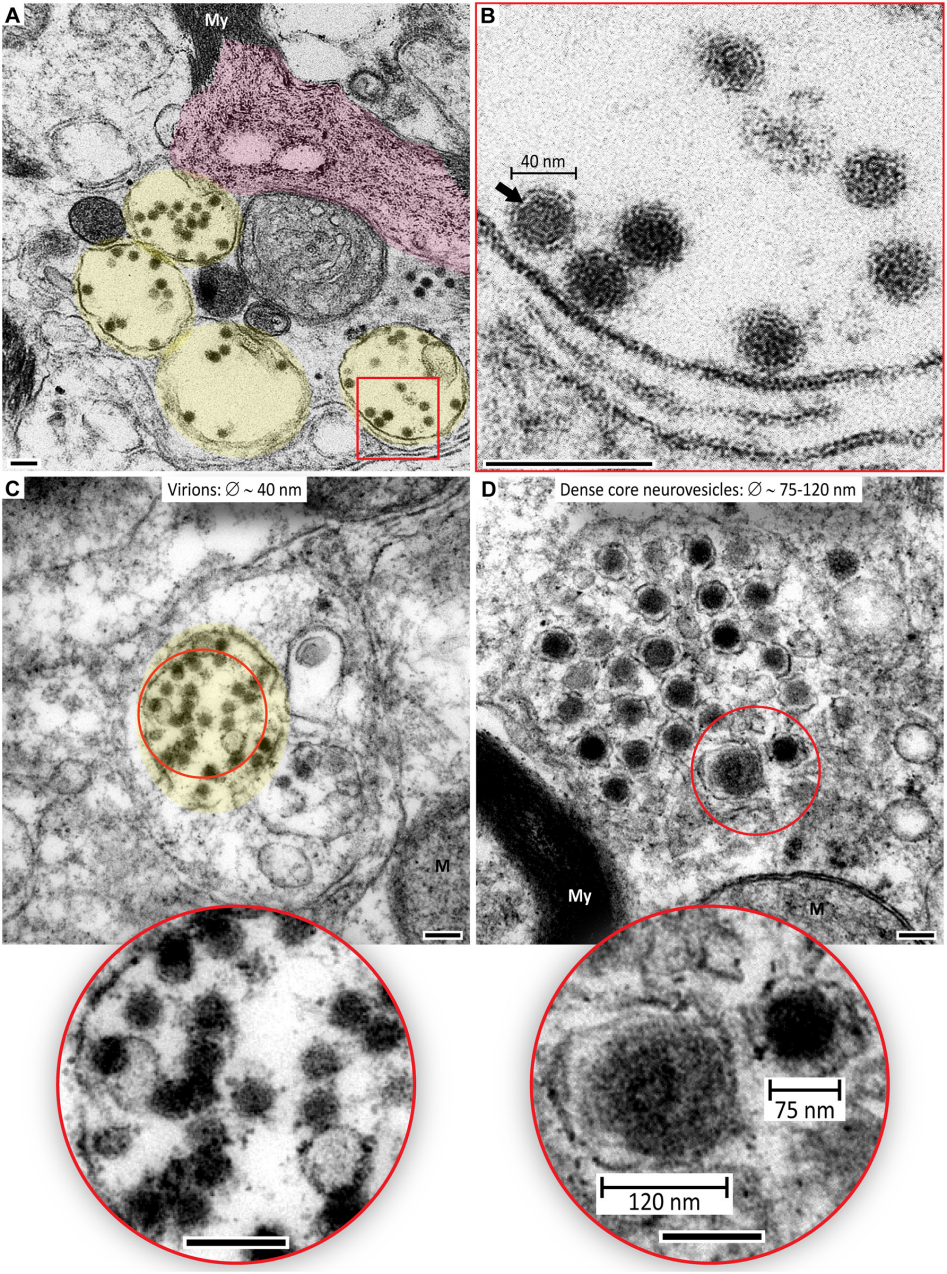
WNV particles versus dense core vesicles in the spinal cord. Shown are electron microscopy images of ventral horns of cervical and lumbar regions of the spinal cord. (**A**) Four large vesicles containing virions are shown by yellow overlays. These vacuoles lie close to a degenerating myelin (My) sheath of axon. Degenerating part of the myelin sheath is shown by magenta overlay. (**B**) Higher magnification image of the boxed area in (**A**) shows several virions of ~ 40 nm in diameter. Note a lighter lipid layer underneath the darker envelope layer especially clearly visible in one of the virions (arrow). (**C** and **D**) Compare two axonal areas containing virions (yellow overlay in **C**) or dense core vesicles (**D**). Insets show red circled areas at higher magnification. Approximate diameters of virions and vesicles are indicated. Scale bars: 100 nm (additional scale bars for dense core vesicles are indicated within the inset in **D**).

Similar to well-defined structures that can be seen in non-polarized cells and have been linked to sites of virus replication [53–55], many infected neurons in this study showed smooth-membrane structures, convoluted membranes, and tubular structures that are characteristic of flavivirus infection. We also observed the formation of prominent paracrystalline arrays (S3 Fig). However, there were several unique findings related exclusively to WNV infection of neurons in this study:

1. Many virions were found within the dendrites of Purkinje cells (Fig 6A–6D). Some virions were seen in close vicinity to the dendritic spines synapsing with axonal terminals.
2. Virions were also found within axons (Fig 7A–7C) and axon terminals (Fig 6E and 6F), often enclosed within vesicles.
3. Some virions were detected within the vesicles with double-layered membrane which most likely represent autophagosomes. Such virion-containing autophagosomes were positioned in axon terminals close to the active zone of the synapse (Fig 6F).

It is also important to note that during EM examination of neurons, the cell organelles such as neuropeptide-containing dense-core vesicles [56] should not be mistaken for virions. This has been also emphasized by other investigators [57]. In this study, we often observed large dense-core vesicles in axons and axon terminals. Two features helped to distinguish between the dense-core vesicles and WNV particles: (i) each dense-core vesicle had a single membrane and (ii) dense-core vesicles were larger in diameter (75–120 nm) compared to WNV virions (40 nm) (compare Fig 7C and 7D, insets).

In human cases of WNV encephalitis, the visualization of WNV particles in neurons by electron microscopy is very rare, likely due to the difficulties in performing a sufficient sampling of particular tissue areas with high virus loads. Interestingly, when found, WNV particles were present in the cerebellar neurons (type of neurons was not specified) [22]. We found virions grouped in the vesicular structures within the dendrites (shafts and spines) as well as within axon terminals in very close vicinity to the synaptic clefts. To our knowledge, this is a first *in vivo* electron microscopy evidence suggesting transsynaptic spread of WNV between synaptically connected neurons in the primate CNS.

Another intriguing ultrastructural finding in this study was that WNV virions in the axon terminals were enclosed in the double-membrane vacuoles indicative of autophagosomes. The role of autophagy in WNV-infected cells *in vitro* is not clear [reviewed in [58]]; however, a recent study in a neonatal mouse model of WNV infection of the CNS showed that pharmacological activation of autophagy by a pro-autophagic peptide can protect against WNV-induced neuronal cell death and improve the clinical outcome [59]. To this end, our finding of WNV virions within the autophagosomes that were positioned pre-synaptically might indicate sequestration of virions for degradation [60] to prevent their transsynaptic release and infection of post-synaptic neuron. Alternatively, since the autophagosomes in neurons are initiated distally at axon terminals and fuse with late endosomes to form the amphisomes that are then transported retrogradely to reach acidic lysosomes in the cell soma [61,62], WNV particles encapsulated within the autophagosomes/amphisomes might take advantage by using retrograde axonal transport to the neuronal perikarya as a way of transneuronal spread. Whether this transport would result in the virion degradation upon fusion with lysosomes or would deliver the virions to the neuronal perikarya for successful subsequent replication remains to be investigated.

Intraneuronal movement of WNV most likely involves microtubules [63–65] and their associated motors for anterograde, retrograde, and/or bidirectional transport [66–69]. In agreement with this scenario, the analysis by electron microscopy in this study revealed virions inside the vesicles that were adjacent to microtubule structures (Fig 6B). Assuming that WNV is transported in neurons within the vesicular structures, it is possible that already established mechanisms for vesicular intraneuronal transport are in use [8]. For example, neuropeptide-containing dense core vesicles can move bidirectionally, switching between anterograde and retrograde axonal transport motors in a conveyor belt-like manner for continuous circulation [70,71]. As mentioned above, it is possible that WNV particles are captured by autophagosome formations at distal axons (whether delivered there by anterograde transport from neuronal soma or transmitted transsynaptically from a post-synaptic neuron) and then delivered by retrograde transport to the neuronal soma for degradation or release and replication.

### Reconstruction of the directionality of transsynaptic virus spread

**Fig 8:**
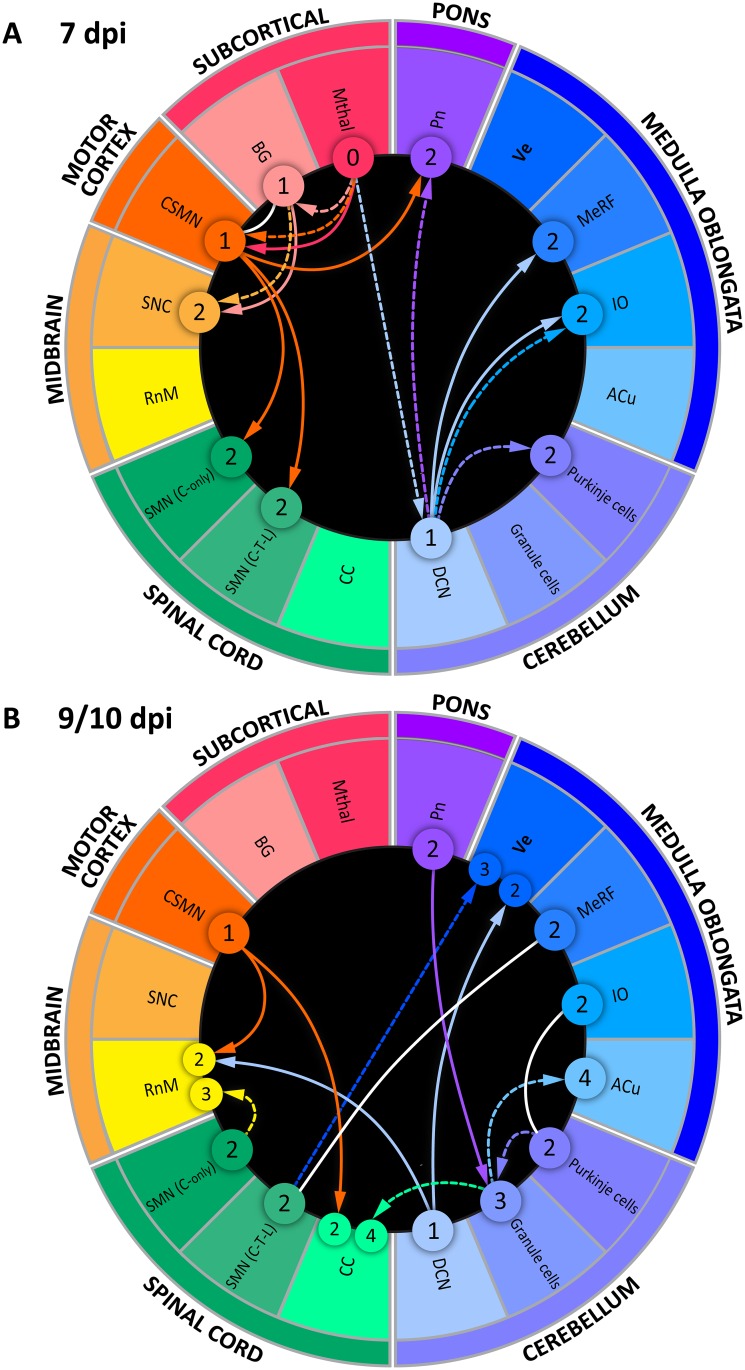
Proposed directionality of WNV spread based on the neuroanatomical connectivity and time of immunohistochemical virus detection. The connectograms illustrate most probable routes and directionality of WNV spread within the CNS in our NHP model of neuroinfection at 7 dpi (**A**) and 9/10 dpi (**B**). Construction and elements of the connectogram are described in Materials and Methods and text. Each arrow has the same color as the structure in the ring from which it originates. The circled numbers 1 to 4 represent most probable “order” of infected neurons within the corresponding anatomical structures. Solid arrows indicate most probable routes of anterograde spread; dashed arrows indicate most probable routes of retrograde spread. White lines indicate the possibility of both, anterograde and retrograde virus spread, due to existence of reciprocal connections between the same orders of neurons. ***Abbreviations*:** Mthal, motor thalamus; BG, basal ganglia; CSMN, corticospinal motor neurons; SNC, substantia nigra pars compacta; RnM, red nucleus magnocellular; SMN, spinal motor neurons (C—cervical; T—thoracic; L—lumbar); CC, Clarke’s column; DCN, deep cerebellar nuclei; ACu, Accessory cuneate nucleus; IO, inferior olivary nuclear complex; MeRF, Medullary reticular formation; Ve, vestibular nuclei; PN, pontine nuclei.

Since the directionality of virus spread cannot be determined based on the “snapshots” revealed by immunohistochemistry and EM, we next attempted to reconstruct the directionality of transsynaptic spread of WNV based on the neuroanatomical connectivity between identified infected structures. For this purpose, we compiled known connectivity information and designed connectograms to capture and visualize the complex neuroanatomical connections between WNV-infected structures in an intuitive and concise way. We created two connectograms (Fig 8) showing a proposed directionality of WNV spread in our model based on the neuroanatomical connectivity and time of immunohistochemical virus detection (i.e., 7 and 9/10 dpi). Reference connectivity information is provided in the S1 Table.

Each connectogram contains two concentric rings and a black core. The names for each major CNS region are given on the outside periphery of outermost ring (i.e., subcortical regions, motor cortex, midbrain, spinal cord, cerebellum, medulla oblongata, and pons) in no particular order. For the outermost ring, each CNS region was assigned a spectrum domain color counterclockwise as follows: subcortical regions (red), motor cortex (orange), midbrain (yellow), spinal cord (green), cerebellum (light blue), medulla oblongata (blue), and pons (purple). The next ring toward the center of the connectogram is divided in sixteen segments. Each segment representing a specific structure/type of neurons includes an abbreviation and is assigned a unique color within the spectrum domain color of the corresponding larger anatomical CNS region. Within the black core, the directions of proposed spread of WNV between the structures/types of neurons are depicted by arrows (anterograde virus spread—solid arrows; retrograde virus spread—dashed arrows; anterograde/retrograde virus spread [due to existence of reciprocal connections]—white lines). Each arrow has the same color as the segment representing a structure/type of neurons from which it originates. The direction of the arrow indicates a proposed direction of virus spread between one neuronal order to the next. The proposed orders of neurons are indicated by circled numbers.

Assuming that the neurons of motor thalamus (Mthal) represent the neuronal order “0” (starter cells), the next neuronal order “1” will be neurons of the deep cerebellar nuclei (DCN), basal ganglia (BG), and corticospinal motor neurons (CSMN) (Fig 8A). The arrows connecting these neuronal groups show that there are three possibilities of the retrograde axonal virus spread (Mthal → DCN; Mthal → BG; and Mthal → CSMN) and one possibility of the anterograde axonal virus spread (Mthal → CSMN). The possibility of both retrograde and anterograde virus spread between Mthal and CSMN exists because of reciprocal connections between these structures. From the neurons of the order “1”, the virus spread to the next neuronal order “2” could also occur by the anterograde axonal transport (BG → SNC; CSMN → SMN; CSMN → Pn; DCN → MeRF; DCN → IO) and by the retrograde axonal transport (BG → SNC; DCN → Pn; DCN → IO; DCN → Purkinje cells).

By the terminal stage of neuroinfection (9/10 dpi; Fig 8B), the directionality of virus spread to the next neuronal orders could occur by anterograde axonal transport (CSMN → RnM; CSMN → CC; DCN → RnM; DCN → Ve; Pn → Granule cells) and by retrograde axonal transport (SMN/Cervical → RnM; SMN → Ve; Purkinje cells → Granule cells; Granule cells → ACu; Granule cells → CC). The maximal neuronal order reached by virus in this model is “4” (ACu and CC).

WNV infection of the neurons of accessory cuneate nucleus (ACu) in the medulla oblongata and Clarke’s column (CC) in the spinal cord is intriguing. These structures relay proprioceptive information from the upper (ACu) and lower (CC) parts of the body to the cerebellum. The axons of these relay neurons terminate as mossy fibers on the granule neurons. It is conceivable that these structures may have been infected by retrograde spread of the virus along the dorsal spinocerebellar tract from granule neurons. However, we found very few WNV-labeled granule neurons in the cerebellum (Fig 4C and 4D). Similarly, the granule cells do not appear to be infected in humans with WNV encephalomyelitis [20]. This is consistent with the recently reported enhanced antiviral response in the granule neurons and might explain their relatively low permissiveness to WNV infection [72]. These considerations suggest a limited contribution of granule neurons to the retrograde spread of WNV along the dorsal spinocerebellar tract to the proprioceptive relay neurons. Recently identified inputs from the descending corticospinal axons [46], better explain the infection of Clarke’s column. By analogy, the infection of the accessory cuneate nucleus could probably be also explained by the cortical inputs (yet to be identified) since this nucleus is the anatomical and functional correlate of Clarke’s column in the medulla.

In summary, our reconstruction of the directionality of WNV spread within the CNS of intrathalamically inoculated NHPs suggests both anterograde and retrograde axonal transport. The connectograms (Fig 8) show eleven possible routes of anterograde and twelve possible routes of retrograde axonal spread (with three unidentifiable directionalities between the same neuronal orders).

Collectively, our results of the anatomical and ultrastructural mapping of WNV neuronal infection in the primate CNS, together with the connectivity-based reconstruction of the directionality of virus spread strongly suggest the following:

1. WNV exclusively infects neurons residing within the specific anatomical structures, each of which plays a major role in the control of movement by the CNS;
2. WNV can spread within the CNS transsynaptically, as demonstrated by our first *in vivo* ultrastructural evidence of the pre- and post-synaptic localization of WNV particles;
3. WNV can spread in both, anterograde and retrograde directions through axonal tracts comprising motor control pathways, which is supported by the connectivity-based reconstruction of virus spread.

Progression of WNV neuroinfection transsynaptically along specific pathways governing motor control in both, anterograde and retrograde directions suggested by this study may open a way for future therapeutic approaches. Although it seems unlikely that antiviral interventions would be justified for asymptomatic or self-limiting WNV cases in humans, we cannot neglect the necessity for development of rational treatments of flavivirus neurological disease. Our findings imply that the focus of such treatments should not only be on limiting virus replication but also on blocking neuron-to-neuron virus transmission, thus preventing further damage to the CNS.

## Supporting Information

S1 Fig Changes in WNV loads within the CNS during the time course of neuroinfection. Radar graphs were constructed using previously reported virus titer data [19] for clarity and to further support current findings. Each radar graph represents the entire CNS counterclockwise from the thalamus (site of inoculation) to the pons/medulla oblongata. This layout was chosen to be similar to the connectogram design (Fig 8). Mean virus titers are shown for each CNS region: (**A**) 3 dpi (n = 3*; no neurological signs*); (**B**) 7 dpi (n = 3; *neurological signs included shaky movements*, *incoordination*, *limb weakness*, *and tremors*); and (**C**) 9/10 dpi (9 dpi [n = 5]; 10 dpi [n = 1]; *moribund state*, *fulminant encephalitis*). The limit of virus detection was 1.7 log_10_ PFU/g of tissue (gray-shadowed in the center of each graph). The motor thalamus (site of virus inoculation) was the major site of virus replication at an earliest time point (3 dpi). Although in lower titers, WNV was also detected in the basal ganglia, motor cortex, and midbrain. Over the course of next 4 days, the virus continued to replicate in the above regions, but the most dramatic increase in virus loads occurred in the remote CNS regions, such as pons/medulla oblongata, cerebellum, and spinal cord. During the next 2–3 days (9/10 dpi), all remaining animals developed a fulminant encephalitis. The pons/medulla oblongata, cerebellum, and spinal cord (including cervical, thoracic, and lumbar regions) were the structures harboring the highest virus loads at the terminal stage of the CNS infection with WNV. Thalamus, cortex, basal ganglia, and midbrain supported WNV replication transiently. The virus loads in these structures returned to their initial levels by 9/10 dpi even though animals succumbed to encephalitis at that time. Thus, after transient replication at the site of inoculation and neighboring structures, WNV had spread to the pons/medulla oblongata, cerebellum, and spinal cord and continued to replicate there.
(TIF)

S2 Fig Cerebellar cortex. WNV antigens are present in the perikarion (yellow arrow), axon (red arrowhead), and dendrites (red arrows) of a Purkinje cell (7 dpi). The underlying granule cells are not labeled in this field. Scale bar: 10 μm.
(TIF)

S3 Fig Representative images of paracrystalline arrays in the neuronal perikarya. Perikarial sites of virus replication/accumulation appear as paracrystalline-like arrays (PCA; outlined in magenta). (**A**) Cerebellar cortex (likely a Purkinje cell) (9 dpi): newly assembled immature virus particles accumulate within the endoplasmic reticulum (ER) creating PCA. (**B**) Another PCA in the cerebellar cortex at higher magnification (9 dpi). One row of the array is outlined by yellow dashed lines and the height of the row (40 nm) is consistent with the approximate size of assembling virions. (**C**) This PCA is situated close to the nucleus in the ventral horn of lumbar spinal cord (likely a spinal motor neuron) (9 dpi). Note a continuity of all shown PCAs with the membranes of endoplasmic reticulum (yellow arrowheads). Nu, nucleus; Nuo, nucleolus. Scale bars: (A) 500 nm; (B, C) 100 nm.
(TIF)

S4 Fig Rarefaction in the granule cell layer of cerebellar cortex in WNV-inoculated monkeys at terminal stage of neuroinfection. Shown is the NeuN immunoreactivity highlighting a normal granule neurons of mock-inoculated animals (**A**) and degenerating granule neurons of WNV-inoculated animals (**B**), both at 9/10 dpi. Note a disappearance of Purkinje cells in **B**. Purkinje cells in **A** appear normal and do not express NeuN. Scale bars (in μm) are shown at the bottom of each image as well as images provided by the magnifier tool of Image Scope (Aperio).
(TIF)

S1 Table Reference information related to the connectogram in Fig 8. (DOCX)
