# Supplementary figures and images for: West Nile Virus Spreads Transsynaptically within the Pathways of Motor Control: Anatomical and Ultrastructural Mapping of Neuronal Virus Infection in the Primate Central Nervous System
Source: PLoS Negl Trop Dis. 2016 Sep 12;10(9):e0004980. doi: 10.1371/journal.pntd.0004980 (PMC5019496; doi:10.1371/journal.pntd.0004980)

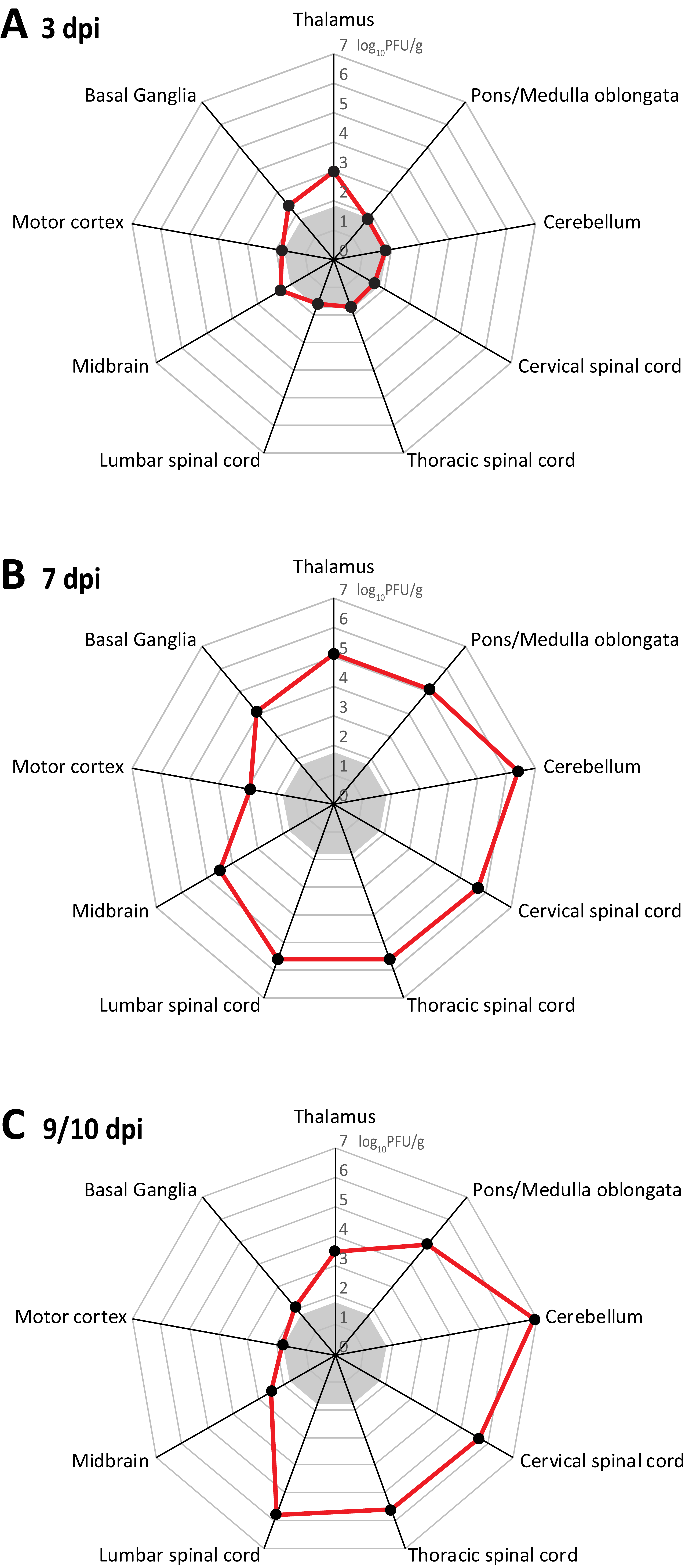

Supplement: S1 Fig — Radar graphs were constructed using previously reported virus titer data [19] for clarity and to further support current findings. Each radar graph represents the entire CNS counterclockwise from the thalamus (site of inoculation) to the pons/medulla oblongata. This layout was chosen to be similar to the connectogram design (Fig 8). Mean virus titers are shown for each CNS region: (A) 3 dpi (n = 3; no neurological signs); (B) 7 dpi (n = 3; neurological signs included shaky movements, incoordination, limb weakness, and tremors); and (C) 9/10 dpi (9 dpi [n = 5]; 10 dpi [n = 1]; moribund state, fulminant encephalitis). The limit of virus detection was 1.7 log10 PFU/g of tissue (gray-shadowed in the center of each graph). The motor thalamus (site of virus inoculation) was the major site of virus replication at an earliest time point (3 dpi). Although in lower titers, WNV was also detected in the basal ganglia, motor cortex, and midbrain. Over the course of next 4 days, the virus continued to replicate in the above regions, but the most dramatic increase in virus loads occurred in the remote CNS regions, such as pons/medulla oblongata, cerebellum, and spinal cord. During the next 2–3 days (9/10 dpi), all remaining animals developed a fulminant encephalitis. The pons/medulla oblongata, cerebellum, and spinal cord (including cervical, thoracic, and lumbar regions) were the structures harboring the highest virus loads at the terminal stage of the CNS infection with WNV. Thalamus, cortex, basal ganglia, and midbrain supported WNV replication transiently. The virus loads in these structures returned to their initial levels by 9/10 dpi even though animals succumbed to encephalitis at that time. Thus, after transient replication at the site of inoculation and neighboring structures, WNV had spread to the pons/medulla oblongata, cerebellum, and spinal cord and continued to replicate there. (TIF) [file pntd.0004980.s001.tif]

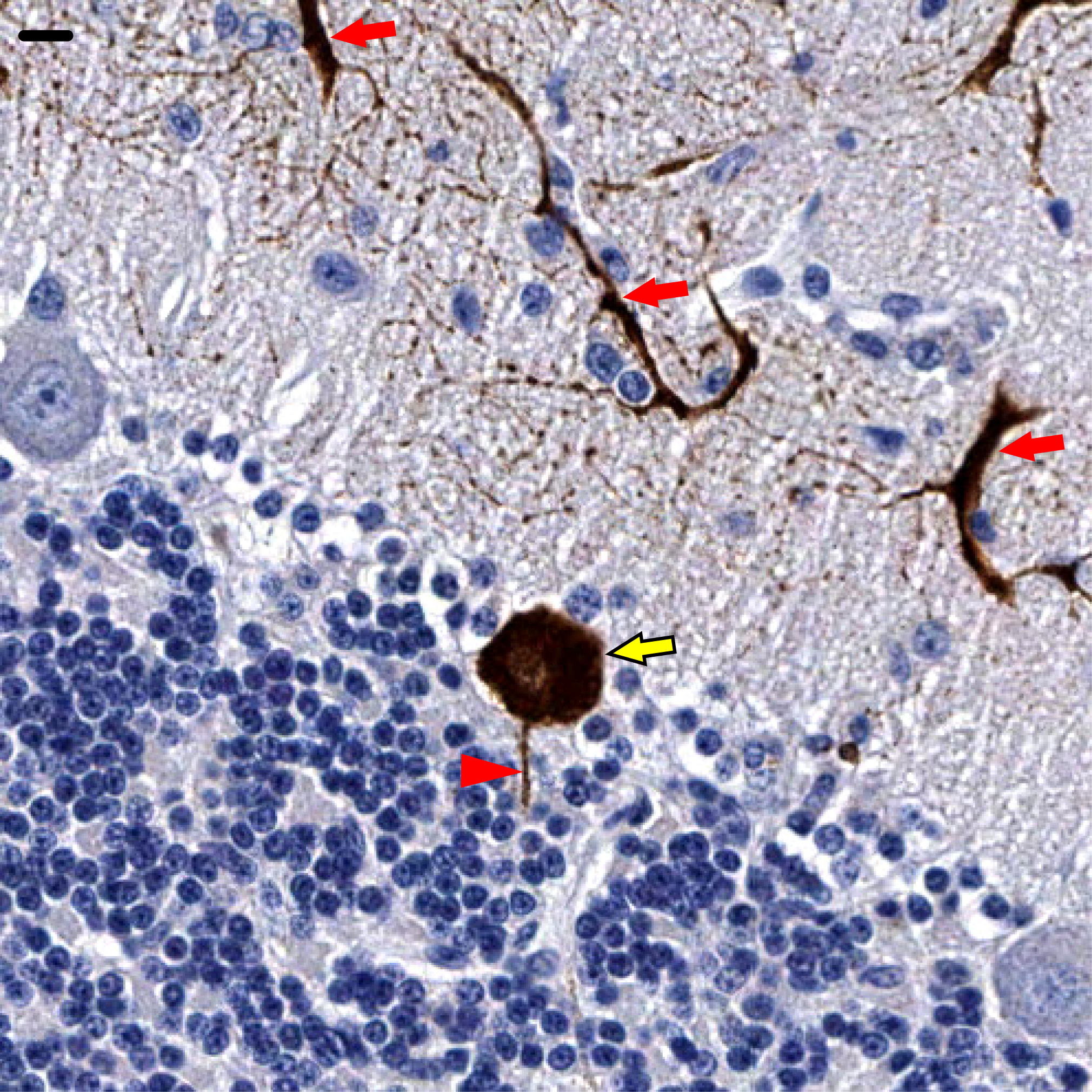

Supplement: S2 Fig — WNV antigens are present in the perikarion (yellow arrow), axon (red arrowhead), and dendrites (red arrows) of a Purkinje cell (7 dpi). The underlying granule cells are not labeled in this field. Scale bar: 10 μm. (TIF) [file pntd.0004980.s002.tif]

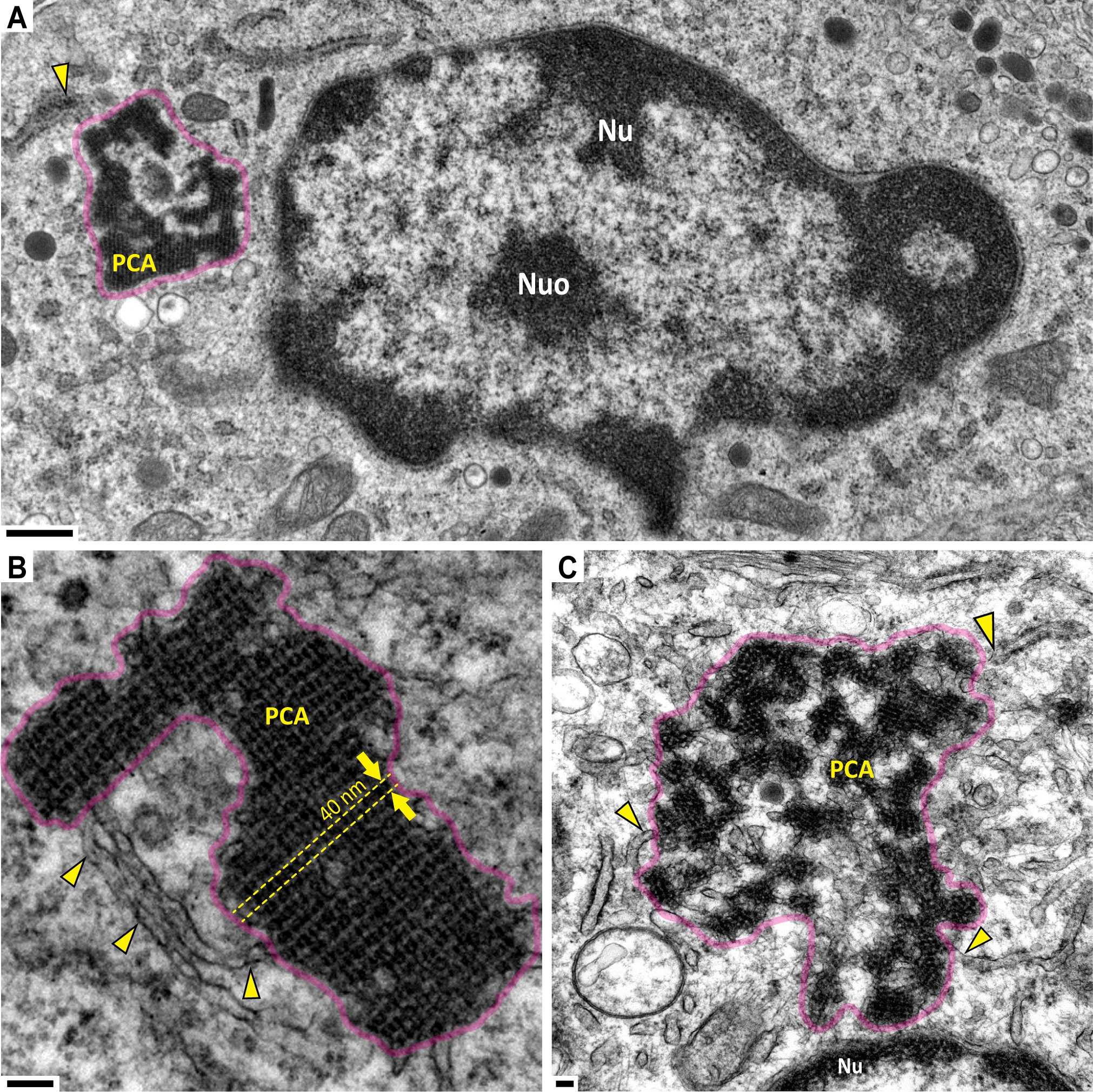

Supplement: S3 Fig — Perikarial sites of virus replication/accumulation appear as paracrystalline-like arrays (PCA; outlined in magenta). (A) Cerebellar cortex (likely a Purkinje cell) (9 dpi): newly assembled immature virus particles accumulate within the endoplasmic reticulum (ER) creating PCA. (B) Another PCA in the cerebellar cortex at higher magnification (9 dpi). One row of the array is outlined by yellow dashed lines and the height of the row (40 nm) is consistent with the approximate size of assembling virions. (C) This PCA is situated close to the nucleus in the ventral horn of lumbar spinal cord (likely a spinal motor neuron) (9 dpi). Note a continuity of all shown PCAs with the membranes of endoplasmic reticulum (yellow arrowheads). Nu, nucleus; Nuo, nucleolus. Scale bars: (A) 500 nm; (B, C) 100 nm. (TIF) [file pntd.0004980.s003.tif]

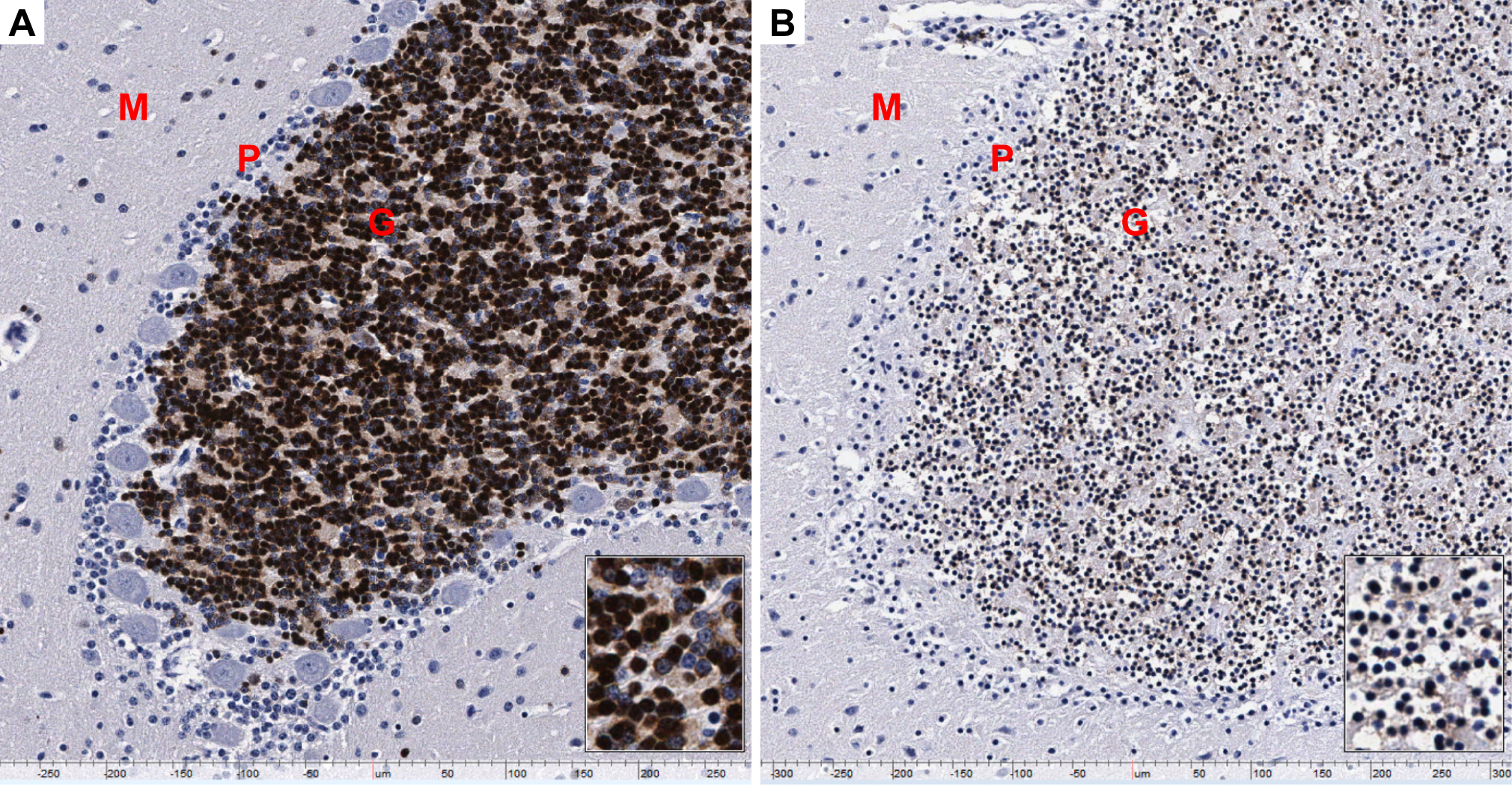

Supplement: S4 Fig — Shown is the NeuN immunoreactivity highlighting a normal granule neurons of mock-inoculated animals (A) and degenerating granule neurons of WNV-inoculated animals (B), both at 9/10 dpi. Note a disappearance of Purkinje cells in B. Purkinje cells in A appear normal and do not express NeuN. Scale bars (in μm) are shown at the bottom of each image as well as images provided by the magnifier tool of Image Scope (Aperio). (TIF) [file pntd.0004980.s004.tif]
