# Supplementary material for: West Nile Virus Spreads Transsynaptically within the Pathways of Motor Control: Anatomical and Ultrastructural Mapping of Neuronal Virus Infection in the Primate Central Nervous System
Source: PLoS Negl Trop Dis. 2016 Sep 12;10(9):e0004980. doi: 10.1371/journal.pntd.0004980 (PMC5019496; doi:10.1371/journal.pntd.0004980)
Supplement: S1 Table — (DOCX) [file pntd.0004980.s005.docx]

**Supplemental Table 1. Reference information related to the connectogram in Figure 8.**

| **WNV-infected structure or type of neurons** | **Abbreviations** | **Neuroanatomical connectivity** | |
| --- | --- | --- | --- |
|  |  | **Input(s) from:** | **Output(s) to:** |
| Corticospinal motor neurons | CSMN | Motor thalamus | Caudate/Putamen  Spinal motor neurons  Red nucleus magnocellular  Pontine nuclei  Clarke’s column |
| Motor thalamus | Mthal | Motor cortex  Basal ganglia  Deep cerebellar nuclei | Motor cortex |
| Basal ganglia | BG | Motor cortex  Substantia nigra pars compacta | Motor thalamus |
| Substantia nigra pars compacta | SNC | Corpus Striatum (BG) (inhibitory) | Corpus Striatum (BG) (dopaminergic) |
| Red nucleus magnocellular | RnM | Motor cortex  Substantia nigra pars reticulata  Deep cerebellar nuclei | Spinal motor neurons |
| Pontine nuclei | Pn | Corticospinal motor neurons | Deep cerebellar nuclei  Granule cells |
| Vestibular nuclei | Ve | Deep cerebellar nuclei | Spinal motor neurons |
| Medullary reticular formation | MeRF | Deep cerebellar nuclei | Spinal motor neurons |
| Inferior olivary nuclear complex | IO | Deep cerebellar nuclei | Deep cerebellar nuclei  Purkinje cells |
| Accessory cuneate nucleus | ACu | N/A [Proprioceptive afferents from upper parts of the body]* | Granule cells |
| Deep cerebellar nuclei | DCN | Purkinje cells  Spinal motor neurons  Inferior olivary nuclei | Motor thalamus  Red nucleus magnocellular  Vestibular nuclei  Medullary Reticular Formation  Inferior olivary nuclei |
| Purkinje cells |  | Inferior olives  Granule cells | Deep cerebellar nuclei |
| Granule cells  (spinocerebellum only – see text) | N/A | Pontine nuclei  Accessory cuneate nucleus  Clarke’s column | Purkinje cells |
| Spinal motor neurons | SMN | Motor cortex  Red nucleus magnocellular  Vestibular nuclei  Medullary Reticular Formation | Neuromuscular junctions |
| Clarke’s column | CC | [Proprioceptive afferents from lower parts of the body]*  Corticospinal motor neurons | Granule cells |

* N/A [Proprioceptive afferents from upper or lower parts of the body] – peripheral nervous system; outside the scope of this study.
